# Rethinking the Appraisal and Approval of Drugs for Fracture Prevention

**DOI:** 10.3389/fphar.2017.00265

**Published:** 2017-05-15

**Authors:** Juan Erviti, Javier Gorricho, Luis C. Saiz, Thomas Perry, James M. Wright

**Affiliations:** ^1^Unit of Drug Assessment, Advice and Research, Navarre Regional Health ServicePamplona, Spain; ^2^Planning, Evaluation and Management Service, Health DepartmentPamplona, Spain; ^3^Department of Anaesthesiology, Pharmacology and Therapeutics, University of British ColumbiaVancouver, BC, Canada

**Keywords:** osteoporosis drugs, hormone replacement therapy, bisphosphonates, calcitonins, teriparatide, denosumab, strontium ranelate, drug regulation

## Abstract

**Background:** In January 2014, the EMA's Pharmacovigilance Risk Assessment Committee recommended that strontium ranelate no longer be used for osteoporosis. However, EMA's Committee for Medicinal Products for Human Use decided to restrict its use rather than ban it. Starting from this fact, evidence of drugs for fracture prevention over the last 30 years was reviewed and lessons to be learnt from this story are highlighted.

**Findings:** The general belief that drug therapy may become a “solution” for fragility fractures is challenged. The key points of the article are as follows:

Lessons 1–5: Bone density and morphometric vertebral compression are not reliable surrogate endpoints. In fact, clinically relevant endpoints are essential to assess harms and benefits in clinical trials. There is a need for assessing overall harm-benefit with well-designed trials, taking into account that drug therapy may not be more effective in high-risk patients. Lessons 6–10: While bisphosphonates and strontium ranelate show a questionable harm-benefit ratio on hip fracture prevention, denosumab results are inconclusive and no benefit has been proved coming from calcitonines or teriparatide. After decades of widespread use, effectiveness of drugs for osteoporosis remains uncertain, yet adverse effects are more apparent.

**Conclusions:** Well-designed and large trials over prolonged follow-up periods, measuring clinically relevant outcomes as hip and other disabling fractures, are urgently needed in order to properly understand the harm-benefit ratio of commonly prescribed drugs. Regulatory agencies should be more transparent and make individual-patient data from all clinical trials publicly available, allowing for independent assessment and pooled analysis.

## Background

The EU expected 3.5 million new fragility fractures in 2010 with an associated cost of 37 billion euros (Svedbom et al., 2013) The hypothesis that increasing bone mineral density (BMD) builds stronger bones made drug therapy the main approach to prevention. But is this still warranted in the light of evidence from randomized trials? Is drug therapy a “solution” for fragility fractures?

In January 2014, the EMA's PRAC recommended that strontium ranelate no longer be used for osteoporosis. However, EMA's CHMP decided to restrict use of strontium ranelate rather than ban it. This is the latest chapter in a disappointing story of drugs that reach “blockbuster” status despite rather than because of scientific evidence. We should learn from the past so that we are not condemned to repeat it.

## Five lessons from trials design

### Lesson 1: bone density is not a reliable surrogate endpoint

By the late 1980's osteoporosis treatments could increase BMD by reducing bone resorption [calcium, calcitonin, or hormone replacement therapy (HRT)] or by stimulating bone formation (sodium fluoride). Fluoride increases BMD more than other drugs. After approval by 8 European regulators, a clinical trial challenged the reputedly positive effects of fluoride on bone fractures (Riggs et al., 1990) Compared with placebo over 4 years, sodium fluoride increased lumbar spine density by 8.2% (95%CI, 5.5–10.9) but caused a three-fold increase in non-vertebral fractures, RR = 3.2 (95%CI, 1.8–5.6). Clinical guidelines no longer recommended it. “Denser” bones may not be stronger bones.

### Lesson 2: morphometric vertebral compression is not a reliable surrogate for clinical fractures

Sodium fluoride did not reduce morphometric vertebral compression (fracture), RR = 0.85 (95%CI, 0.6–1.2). Compression of a vertebra visualized by X-ray is not necessarily a clinical fracture. The deleterious effects of sodium fluoride on non-vertebral fractures could not be anticipated from data on BMD or morphometric vertebral compression.

Reductions in vertebral height between 20 and 25% at any point of the vertebrae have been defined for osteoporosis research purposes as “fractures.” However, they are not abnormalities typically considered in clinical practice as relevant to health. The inventors of this “fracture” definition acknowledged its serious limitations, warning that “fracture diagnosis could be arbitrary to some extent” (Genant et al., 1993). Some clinical trials have altered the definition. For example, in trials of risedronate and strontium ranelate “fracture” was arbitrarily redefined as a decrease in vertebral height of at least 15%. This artificially increased the reported “incidence” of “vertebral fractures.”

Only one third of all vertebral “fractures” diagnosed radiologically are symptomatic. The only osteoporosis drug trials reporting quality of life were the FIT1 and FIT2 trials of alendronate. At enrolment, 70% of women in FIT1 and 35% in FIT2 were diagnosed with vertebral “fracture,” yet 93 and 95% of women considered their self-rated health status “good, very good, or excellent” (Ware and Gandek, 1998). What was the clinical relevance of morphometric vertebral “fractures” diagnosed at baseline?

### Lesson 3: clinically relevant endpoints are essential to assess harms and benefits in RCT

For treatment of osteoporosis, hip fracture is the most relevant endpoint other than total mortality. “Non-vertebral fractures” are often presented as a clinically important variable, but this raises a number of problems. Their definition varies between trials, which may facilitate “cherry-picking” of reported outcomes if the definition was not pre-specified in the trial protocol and followed scrupulously in reporting. This composite endpoint may include clinically important events (e.g., symptomatic vertebral compressions), but also inconsequential radiologically determined compressions or other minor fractures. In many trials, non-vertebral fracture outcomes are discordant with the observed incidence of hip fractures.

Bisphosphonate effects on hip fracture are disappointing. Most trials comparing alendronate, risedronate, and ibandronate with placebo showed no significant reduction of hip fractures. Meta-analysis of alendronate and risedronate compared with placebo identifies a statistically significant decrease in hip fracture incidence, but the effect size is very small and 75% of trials had a high or unclear risk of bias. The small apparent reduction in hip fracture may not be real, or is at best an exaggeration of the real benefit (Therapeutics Initiative, 2011; see Supplementary Material).

### Lesson 4: assess overall harm-benefit with well-designed trials

When HRT was launched, evidence that it prevents fractures was far from compelling. Observational studies showed a decreased fracture incidence, and hypothetical cardiovascular benefits were attributed to HRT on the basis of an “improvement” in lipid profile. The first large trial (Rossouw et al., 2002) of HRT in postmenopausal women included 16,608 women aged 50–79 years, with a scheduled follow-up of some 8 years. It was stopped in 2002 after 5.2 years. Five fewer hip fractures per 10,000 person-years [HR = 0.66 (95% CI, 0.45–0.98)] and 6 fewer colorectal cancers per 10,000 person-years were observed. However, coronary heart disease, stroke, pulmonary embolism, and invasive breast cancer all increased. With a net health effect of 19 serious adverse events per 10,000 person-years, HRT's unfavorable harm-benefit ratio no longer appealed as “first-line” therapy.

Clinical trials evaluating harm-benefit balance in osteoporosis or fracture prevention should be well-powered long-term studies that include hard endpoints. Total mortality, total serious adverse events, hip fractures, and functional status are essential outcomes.

### Lesson 5: drug therapy may not be more effective in high-risk patients

Aging is the main risk factor for fractures. However, there is no evidence that osteoporosis drugs perform better in elderly people. The European Public Assessment Report on zoledronic acid noted a marginal reduction in relative risk for hip fractures in patients ≥75 years (18%) compared with younger patients in Study 2301. Age subgroups pre-specified in the statistical analysis plan (<70 years; 70–74 years, and ≥75 years) included over 1,100 women each. Observed hip fracture risk increased with age in the zoledronic acid group compared with placebo, although the trend did not reach statistical significance. These results do not support the hypothesis that bisphosphonates might be more effective in high-risk populations.

A single large trial compared the effect of risedronate vs. placebo on hip fracture as the primary endpoint, depending on age (McClung et al., 2001). The mean age of women age 70–79 with osteoporosis (*n* = 5,445) was 74 years, vs. 83 years in women >80 with at least one risk factor for hip fracture (*n* = 3,886). In the older higher risk women, the placebo group had a higher total fracture risk (5.1%) than in the younger population (3.2%). However, risedronate did not reduce hip fractures in the older patient group: 4.2% with risedronate vs. 5.1% with placebo, RR = 0.8 (95% CI, 0.6–1.2).

## Five additional lessons from specific drugs:

### Lesson 6: bisphosphonates

Demonstrating increased BMD led to approval in the 1990s. Although no information about their effects on fracture risk was available at commercialization, millions of women began treatment.

The longest clinical trial was published in 2006 (Black et al., 2006). Of 3,236 women previously randomized to 5 years of alendronate, one third were re-randomized to continue alendronate (5 or 10 mg/d) or to receive placebo for another 5 years. Continuing alendronate decreased clinically recognized vertebral fractures [alendronate 2.4% vs. placebo 5.3% RR 0.45 (0.24–0.85)], but not total clinical fractures [alendronate 19.9% vs. placebo 21.3%, RR 0.93 (0.71–1.21)], nor hip fractures.

FDA researchers analyzed these results along with long-term extension trials of risedronate and zoledronic acid. Pooled data from patients who received continuous bisphosphonate treatment for 6 or more years indicated total fracture rates ranging from 9.3 to 10.6%, whereas the rates for patients switched to placebo were 8.0–8.8%. This suggests that long-term use of bisphosphonates is not beneficial and may be harmful (Whitaker et al., 2012).

In 2008, a cohort study in Danish women with no previous hip fracture was published. The incidence of hip fractures increased by 45% (relative) in the group treated with alendronate, equivalent in absolute risk increase to 6 cases per 1,000 woman–years (Abrahamsen et al., 2009). Updated information confirmed the results (Abrahamsen et al., 2010). Both groups were matched by sex, age, and location of baseline fracture. However, bisphosphonates could have been prescribed preferentially to patients at higher risk for fractures leading to indication bias. Apart from matching the two cohorts, results were also adjusted by number of comedications, Charlson comorbidity index, and use of any oral glucocorticoid within the last year. Though residual confounding may still exist after adjustment, it is rather unlikely that this may account for the 45% increase in hip fracture observed, and let alone reverse a hypothetical protective effect of alendronate on hip fractures.

A similar Swedish study found a significant 41 and 19% relative risk increase in trochanteric and femoral-neck fracture, in bisphosphonate users after adjustment. The absolute increase in fracture incidence was 3.4 and 3.3 cases per 1,000 women–years (Schilcher et al., 2011).

In Spain, a case-control study evaluated the association between long-term use of bisphosphonates and risk of hip fracture, compared with women 65 years or older who had never used bisphosphonates. Oral bisphosphonates were not associated with a decreased risk of hip fracture. We found a statistically significant increased incidence of hip fracture in patients exposed to bisphosphonates for >3 years, compared with women at <3 years exposure (Erviti et al., 2013).

All three observational studies found no benefit of bisphosphonate use for hip fracture risk, but a numerically higher incidence of hip fracture. Despite adjustment for confounding factors, residual indication bias may remain, but the results call into question the effectiveness of bisphosphonates to prevent hip fractures (Table 1).

**Table 1:** Real-life evidence^*^ on the effectiveness of bone targeted pharmacotherapy.

**Authors**	**Journal**	**Year**	**Study sample**	**Set-up/Design**	**General finding**	**Strengths**	**Weaknesses**
Abrahamsen et al.	*J. Bone Miner. Res*.	2009	A register-based national cohort study	Matched cohort study in patients with prior non-hip fractures	Hip fracture risk increased in alendronate users vs. untreated controls RR = 1.45 (1.21–1.74)	Long-term follow-up (8 years)	Includes alendronate only
Abrahamsen et al.	*J. Clin. Endocr. Metab*.	2010	A register-based national cohort analysis	Fracture rates in a cohort of patients who began alendronate between 1996 and 2005 compared with age- and sex-matched control subjects from the background population	Hip fracture risk (neck or pertrochanteric) increased in alendronate users vs. untreated controls. Women, HR = 1.37 (1.30–1.46) Men, HR = 2.47 (2.07–2.95)	Long-term follow-up. Mean follow-up, 3.4 years; 34,204 patients provided 5–10 years of follow-up, and 1,920 subjects provided over 10 years of follow-up. Differentiated results in men and women are available.	Includes alendronate only
Schilcher et al.	*N. Engl. J. Med*.	2011		Population-based nationwide cohort analysis (Sweden)	Bisphosphonate users increased the risk of hip fractures vs. non-users, both femoral neck fractures (RR = 1.19 (1.09–1.29) and trochanteric fractures (RR = 1.41 (1.29–1.54)	Large sample size (over 10,000 hip fractures)	Short follow-up period
Erviti et al.	*BMJ Open*	2013	General practice research database operated by the Spanish Medicines Agency	Case–control study nested in a cohort	Bisphosphonate use was not associated with decreased risk of hip fracture in women aged 65 or older as compared to never use, OR = 1.09 (95% CI, 0.94–1.27). Increased risk for hip fracture was observed in patients exposed to bisphosphonates over 3 years since first prescription (p for trend = 0.03).	Carried out in a Mediterranean population (low fracture risk)	Short follow-up period

* *Inclusion criteria: observational studies assessing the effects of bisphosphonates vs. non-users or placebo in the incidence of hip fractures in primary prevention*.

Years after approval of bisphosphonates, an increased incidence of osteonecrosis of the jaw was detected, as well as atypical femoral fractures and bone pain. Bisphosphonates have other important adverse effects such as esophageal damage.

Despite their questionable harm-benefit ratio, bisphosphonates are still widely promoted and prescribed even after US FDA officers pointed out a possible increase in fracture risk in women receiving bisphosphonates (Whitaker et al., 2012). A recent study showed the rational and mechanism by which bisphosphonates may cause microcrack accumulation, leading to a loss of microstructural integrity and consequently, reduced mechanical strength (Ma et al., 2017).

### Lesson 7: calcitonins

These were amongst the first drugs used for osteoporosis. Their use flourished after marketing in the 1980s, even though calcitonins were never proven effective for clinically important endpoints. Recently, a signal of increased cancer risk was detected and the EMA^1^ concluded that “*the benefits of calcitonin-containing medicines do not outweigh their risks in the treatment of osteoporosis and they should no longer be used for this condition*.”

### Lesson 8: teriparatide

In 2003, teriparatide (rhPTH) was authorized in the European Union. This drug reduced morphometric vertebral “fractures,” but not hip fracture. Rat studies indicated a long term risk for osteosarcoma, so the recommended treatment is limited to <2 years. In the European database of suspected adverse drug reaction (ADR) reports, the most frequent suspected ADR to January 2016 are musculoskeletal and connective tissue disorders (3,943 cases), along with a surprising number of cancer. Thirteen years after approval of teriparatide, its long term safety profile remains unclear and no clinical trial has addressed this issue.

### Lesson 9: denosumab

This monoclonal antibody also reduces morphometric vertebral “fractures.” Data on prevention of hip fractures are inconclusive^2^. The only trial of denosumab in postmenopausal women with osteoporosis presents a high risk of bias. In addition, serious irregularities found during trial inspections raise concern about the veracity of published data^3^.

### Lesson 10: strontium ranelate

This drug was never approved by the FDA, but after its 2004 European “launch,” strontium flew below the radar until falling from grace in 2014. It reduces morphometric vertebral “fractures,” but has questionable, if any, benefits for hip fracture prevention (Meunier et al., 2004; Reginster et al., 2005). Effects on total non-vertebral fractures were barely statistically significant, and depend on inclusion of rib and sternal fractures in the composite endpoint. These fractures are not normally considered as osteoporotic.

## Conclusions

After decades of widespread use, effectiveness of drugs for osteoporosis remains uncertain, yet adverse effects are more apparent.

Without well-designed large trials that measure clinically relevant outcomes over prolonged follow-up periods, we may wait 30 years to find out that a drug has no net benefit. This was true for HRT, calcitonin, and probably strontium ranelate. Meanwhile millions of people are being exposed to drugs for which we do not understand the benefit to harm ratio.

We need effectiveness evidence for clinically important endpoints such as hip and other disabling fractures. BMD or morphometric vertebral “fractures” were never a substitute other than for marketing-based medicine.

The EMA is well aware that its osteoporosis guidelines need to be reviewed^4^ but no new guideline is available. Regulatory agencies should be transparent and make individual-patient data from all clinical trials publicly available, to allow for independent assessment and pooled analysis.

## Author contributions

JE conceived and drafted the article. JG, LS, TP, and JW revised the draft critically, adding significant input. All the authors (JE, JG, LS, TP, and JW) gave final approval for the last version and agreed to be accountable for all aspects of the published work.

### Conflict of interest statement

The authors declare that the research was conducted in the absence of any commercial or financial relationships that could be construed as a potential conflict of interest.

## Footnotes

^1^Committee for Medicinal Products for Human Use. European Medicines Agency (2013) *Assessment Report for Calcitonin Containing Medicinal Products*. Report No.: EMA/109665/2013. Available online at: www.ema.europa.eu/docs/en_GB/document_library/Referrals_document/Calcitonin_31/WC500146172.pdf (Accessed January 11, 2016).

^2^Center for Drug Evaluation and Research. Food and Drug Administration. *Prolia Statistical Review*. Available online at: http://www.accessdata.fda.gov/drugsatfda_docs/nda/2010/125320s000StatR.pdf (Accessed January 11, 2016).

^3^Committee for Medicinal Products for Human Use. European Medicines Agency (2010). *Assessment Report for Prolia*. Report No.: EMEA/H/C/001120. Available online at: http://www.ema.europa.eu/docs/en_GB/document_library/EPAR_-_Public_assessment_report/human/001120/WC500093529.pdf (Accessed January 11, 2016).

^4^Committee for Medicinal Products for Human Use. European Medicines Agency (2012) *Concept Paper on the Need for Revision of the Guideline on the Evaluation of Medicinal Products in the Treatment of Primary Osteoporosis*. Report No.: EMA/CHMP/520786/2012. Available online at: www.ema.europa.eu/ema/pages/includes/document/open_document.jsp?webContentId=WC500134467 (Accessed December 10, 2014).
