# Supplementary material for: Rethinking the Appraisal and Approval of Drugs for Fracture Prevention
Source: Front Pharmacol. 2017 May 15;8:265. doi: 10.3389/fphar.2017.00265 (PMC5430022; doi:10.3389/fphar.2017.00265)
Supplement: Supplementary file 1 [file Table2.DOCX]

**Table 2.** List of all included studies in ‘Therapeutics Letter 83; 2011 Sept-Oct 83:1-2 which provided clinical outcome data [reference in article Num. 5].

| 1. Adami S, Passeri M, Ortolani S, Broggini M, Carratelli L, Caruso I, et al. Effects of oral alendronate and intranasal salmon calcitonin on bone mass and biochemical markers of bone turnover in postmenopausal women with osteoporosis. Bone (1995) 17(4):383–90. |
| --- |
| 1. Ascott-Evans BH, Guanabens N, Kivinen S, Stuckey BG, Magaril CH, Vandormael K, et al. Alendronate Prevents Loss of Bone Density Associated With Discontinuation of Hormone Replacement Therapy. Arch Intern Med (2003) **163**:789-794. |
| 1. Bell NH, Bilezikian JP, Bone HG III, Kaur A, Maragoto A, Santora AC, Study Group. Alendronate increases bone mass and reduces bone markers in postmenopausal African-American women. Journal of Clinical Endocrinology & Metabolism (2002) **87**(6):2792–7. |
| 1. Black DM, Cummings SR, Karpf DB, Cauley JA, Thompson DE, Nevitt MC et al. Randomised trial of effect of alendronate on risk of fracture in women with existing vertebral fractures. Fracture Intervention Trial Research Group. Lancet (1996) **348**: 1535-1541. |
| 1. Bone HG, Downs RW Jr, Tucci JR, Harris ST, Weinstein RS, Licata AA, et al. Dose-response relationships for alendronate treatment in osteoporotic elderly women. Alendronate Elderly Osteoporosis Study Centers. Journal of Clinical Endocrinology & Metabolism (1997) **82**(1):265-74. |
| 1. Bone HG, Greenspan SL, McKeever C, Bell N, Davidson M, Downs RW, et al. Alendronate and estrogen effects in postmenopausal women with low bone mineral density. Alendronate/Estrogen Study Group. Journal of Clinical Endocrinology & Metabolism (2000) **85**(2):720–6. |
| 1. Cecelia D, Jodar E, Fernandez C, Resines C, Hawkins F. Effect of alendronate in elderly patients after low trauma hip fracture repair. Osteoporos Int (2009) **20**: 903-9102009 |
| 1. Chailurkit LO, Jongjaroenprasert W, Rungbunnapun S, Ongphiphadhanakul B, Sae-tung S, Rajatanavin R. Effect of alendronate on bone mineral density and bone turnover in Thai postmenopausal osteoporosis. Journal of Bone & Mineral Metabolism (2003) **21**(6):421–7. |
| 1. Chesnut CH III, McClung MR, Ensrud KE, Bell NH, Genant HK, Harris ST, et al. Alendronate treatment of the postmenopausal osteoporotic woman: effect of multiple dosages on bone mass and bone remodeling. American Journal of Medicine (1995) **99**(2):144-52. |
| 1. Clemmesen B, Ravn P, Zegels B, Taquet AN, Christiansen C, Reginster JY. A 2 year phase II strudy with a1 year follow up of risedronate (NE 58095) in post-menopausal osteoporosis. Osteoporos Int (1997) **7**:488–495. |
| 1. Cummings SR, Black DM, Thompson DE, Applegate WB, Barrett-Connor E, Musliner TA, et al. Effect of alendronate on risk of fracture in women with low bone density but without vertebral fractures: results from the Fracture Intervention Trial. JAMA (1998) **280**: 2077-2082. |
| 1. Dobnig H, Hofbauer LC, Viereck V, Obermayer-Pietsch B, Fahrleitner-Pammer A, Dobnig H, et al. Changes in the RANK ligand/osteoprotegerin system are correlated to changes in bone mineral density in bisphosphonate-treated osteoporotic patients. Osteoporosis International (2006) **17**(5):693-203. |
| 1. Dursun N, Dursun E, Yalcin S. Comparison of alendronate, calcitonin and calcium treatments in postmenopausal osteoporosis. International Journal of Clinical Practice (2001) **55**(8):505-9. |
| 1. Fogelman I, Ribot C, Smith R, Ethgen D, Sod E, Reginster JY. Risedronate reverses bone loss in postmenopausal women with low bone mass: results from a multinational, double-blind, placebo-controlled trial. BMD-MN Study Group. Journal of Clinical Endocrinology & Metabolism (2000) **85**(5):1895-900. |
| 1. Gurlek A, Bayraktar M, Gedik O. Comparison of calcitriol treatment with etidronate-calcitriol and calcitonin-calcitriol combinations in Turkish women with postmenopausal osteoporosis: a prospective study. Calciﬁed Tissue International (1997) **61**(1):39–43. |
| 1. Harris ST, Watts NB, Genant HK, McKeever CD, Hangartner T, Keller M, et al. (1999) Effects of risedronate treatment on vertebral and nonvertebral fractures in women with postmenopausal osteoporosis: a randomized controlled trial. Vertebral Efficacy With Risedronate Therapy (VERT) Study Group. JAMA (1999) **282**(14):1344-52 |
| 1. Hosking D, Adami S, Felsenberg D, Andia JC, Valimaki M, Benhamou L, et al. Comparison of change in bone resorption and bone mineral density with once-weekly alendronate and daily risedronate: a randomised, placebo-controlled study. Current Medical Research & Opinion (2003) **19**(5):383–94. |
| 1. Ishida Y, Kawai S. Comparative efficacy of hormone replacement therapy, etidronate, calcitonin, alfacalcidol, and vitamin K in postmenopausal women with osteoporosis: The Yamaguchi Osteoporosis Prevention Study. American Journal of Medicine (2004) **117**(8):549-55. |
| 1. Johnell O, Scheele WH, Lu Y, Reginster JY, Need AG, Seeman E. Additive effects of raloxifene and alendronate on bone density and biochemical markers of bone remodeling in postmenopausal women with osteoporosis. [see comment]. Journal of Clinical Endocrinology & Metabolism (2002) **87**(3):985–92. |
| 1. Lau EM, Woo J, Chan YH, Griffith J. Alendronate prevents bone loss in Chinese women with osteoporosis. Bone (2000) **27**(5):677–80. |
| 1. Liberman UA, Weiss SR, Broll J, Minne HW, Quan H, Bell NH, et al. Effect of oral alendronate on bone mineral density and the incidence of fractures in postmenopausal osteoporosis. The Alendronate Phase III Osteoporosis Treatment Study Group. [see comment]. New England Journal of Medicine (1995) **333**(22):1437-43. |
| 1. Lyritis GP, Paspati TI, Skarantavos Gr, Androulakis GC. The effect of a modified etidronate cyclical regimen on post-menopausal Osteoporosis: A four year study. Clinical Rheumatology (1997) **16**(4): 354-360., |
| 1. McClung MR, Geusens P, Miller PD, Zippel H, Bensen WG, Roux C, et al. Effect of risedronate on the risk of hip fracture in elderly women. NEJM (2001) **344**: 333-40. |
| 1. Montessori ML, Scheele WH, Netelenbos JC, Kerkhoff JF, Bakker K. The use of etidronate and calcium versus calcium alone in the treatment of postmenopausal osteopenia: results of three years of treatment. Osteoporosis International (1997) **7**(1):52-8. |
| 1. Pols HA, Felsenberg D, Hanley DA, Stepan J, Munoz-Torres M, Wilkin TJ, et al. Multinational, placebo-controlled, randomized trial of the effects of alendronate on bone density and fracture risk in postmenopausal women with low bone mass: results of the FOSIT study. Fosamax International Trial Study Group. Osteoporos Int (1999) **9**: 461-468. |
| 1. Qin Ling, Choy W, Au S, Fan M, Leung P. Alendronate increases BMD at appendicular and axial skeleton in patients with established osteoporosis. Journal of Orthopaedic Surgery and Research (2007) **2**:9 doi:10.1186/1749-799X-2-9. |
| 1. Reginster J, Minne HW, Sorensen OH, Hooper M, Roux C, Brandi ML, et al. Randomized trial of the effects of risedronate on vertebral fractures in women with established postmenopausal osteoporosis. Vertebral Efficacy with Risedronate Therapy (VERT) Study Group. Osteoporosis International (2000) **11**(1):83-91. |
| 1. Rhee Y, Kang M, Min Y, Byun D, Chung Y, Ahn C, et al. Effects of a combined alendronate and calcitriol agent (Maxmarvil) on bone metabolism in Korean postmenopausal women: a multicenter, double-blind, randomized, placebo-controlled study. Osteoporosis International (2006) **17**(12):1801-7. |
| 1. Shiota E, Tsuchiya K, Yamaoka K, Kawano O. Effect of intermittent cyclical treatment with etidronate disodium (HEBP) and calcium plus alphacalcidol in postmenopausal osteoporosis. Journal of Orthopaedic Science (2001) **6**(2):133-6. |
| 1. Storm T, Thamsborg G, Steiniche T, Genant HK, Sorensen OH. Effect of intermittent cyclical etidronate therapy on bone mass and fracture rate in women with postmenopausal osteoporosis [see comment]. New England Journal of Medicine (1990) **322**(18):1265-71. |
| 1. Watts NB, Harris ST, Genant HK, Wasnich RD, Miller PD, Jackson RD, et al. Intermittent cyclical etidronate treatment of postmenopausal osteoporosis [see comment]. New England Journal of Medicine (1990) **323**(2):73-9. |
| 1. Wimalawansa SJ. A four-year randomized controlled trial of hormone replacement and bisphosphonate, alone or in combination, in women with postmenopausal osteoporosis. American Journal of Medicine (1998) **104**(3):219-26. |
| 1. Yan Y, Wang W, Zhu H, Li M, Liu J, Luo B, et al. The efficacy and tolerability of once-weekly alendronate 70 mg on bone mineral density and bone turnover markers in postmenopausal Chinese women with osteoporosis. J Bone Miner Metab (2009) **27**:471–478. |
